# Health Impact of Fasting in Saudi Arabia during Ramadan: Association with Disturbed Circadian Rhythm and Metabolic and Sleeping Patterns

**DOI:** 10.1371/journal.pone.0096500

**Published:** 2014-05-08

**Authors:** Ghada M. Ajabnoor, Suhad Bahijri, Anwar Borai, Altaf A. Abdulkhaliq, Jumana Y. Al-Aama, George P. Chrousos

**Affiliations:** 1 Department of Clinical Biochemistry, Faculty of Medicine, King Abdulaziz University, Jeddah, Saudi Arabia; 2 Saudi Diabetes Study Research Group, King Fahd Medical Research Center, King Abdulaziz University, Jeddah, Saudi Arabia; 3 King Abdullah International Medical Research Center (KAIMRC), College of Medicine, King Saud Bin Abdulaziz University for Health Sciences (KSAU-HS), Jeddah, Saudi Arabia; 4 Department of Clinical Biochemistry, Faculty of Medicine, Umm Al-Qura University, Makkah, Saudi Arabia; 5 Department of Genetic Medicine, Faculty of Medicine, King Abdulaziz University, Jeddah, Saudi Arabia; 6 Princess Al-Jawhara Al Brahim Center of Excellence in Research of Hereditary Disorders, King Abdulaziz University, Jeddah, Saudi Arabia; 7 First Department of Pediatrics, University of Athens Medical School, “Aghia Sophia” Children’s Hospital, Athens, Greece; University of Kansas Medical Center, United States of America

## Abstract

**Background:**

Muslims go through strict Ramadan fasting from dawn till sunset for one month yearly. These practices are associated with disturbed feeding and sleep patterns. We recently demonstrated that, during Ramadan, circadian cortisol rhythm of Saudis is abolished, exposing these subjects to continuously increased cortisol levels.

**Hypothesis:**

Secretory patterns of other hormones and metabolic parameters associated with cortisol, and insulin resistance, might be affected during Ramadan.

**Protocol:**

Ramadan practitioners (18 males, 5 females; mean age ±SEM = 23.16±1.2 years) were evaluated before and two weeks into Ramadan. Blood was collected for measurements of endocrine and metabolic parameters at 9 am (±1 hour) and again twelve hours later.

**Results:**

In Ramadan, glucose concentration was kept within normal range, with a significant increase in the morning. Mean morning concentration of leptin was significantly higher than pre-Ramadan values (p = 0.001), in contrast to that of adiponectin, which was significantly lower (p<0.001). These changes were associated with increased insulin resistance in morning and evening. Concentrations of hsCRP were lower during Ramadan than those during regular living conditions, however, normal circadian fluctuation was abolished (p = 0.49). Even though means of liver enzymes, total bilirubin, total protein and albumin were all decreased during Ramadan, statistically lower means were only noted for GGT, total protein, and albumin (p = 0.018, 0.002 and 0.001 respectively).

**Discussion:**

Saudi Ramadan practitioners have altered adipokine patterns, typical of insulin resistance. The noted decreases of hsCRP, liver enzymes, total protein, and albumin, are most likely a result of fasting, while loss of circadian rhythmicity of hsCRP is probably due to loss of circadian cortisol rhythm.

**Conclusions:**

Modern Ramadan practices in Saudi Arabia, which are associated with evening hypercortisolism, are also characterized by altered adipokines patterns, and an abolished hsCRP circadian rhythm, all likely to increase cardiometabolic risk.

## Introduction

The combined inputs of the master circadian clock in the suprachiasmatic nucleus (SCN) of the hypothalamus and of the rhythmic behaviors of the rest/sleep *vs.* wake and feeding *vs.* fasting states control the overall temporal organization of the neuroendocrine system, as well as other behavioral and physiologic systems, along the 24-hour day [1], [2]. Conversely, the peripheral clocks and the above rhythmic states may influence neuroendocrine tempos independently of the direct control of the central circadian clock [3]. The overwhelming evidence that the circadian and metabolic systems are linked together at molecular, cellular and behavioral levels has fueled great interest in the possible roles of circadian disorganization in obesity, diabetes and other cardiometabolic disorders [4]–[6].

Adequate sleep duration is vital for cardiovascular health, while inadequate duration of quality sleep is specifically associated with increased cardiovascular morbidity [7]–[9]. On the other hand, intermittent fasting (IF; reduced meal frequency) and caloric restriction (CR) enhance cardiovascular and brain functions and improve several risk factors for coronary artery disease and stroke, including a reduction in blood pressure and increased insulin sensitivity [10]. Therefore, the obligatory fasting from dawn to sunset during the month of Ramadan for all healthy Muslims is believed to bring health benefits. Indeed, studies on Ramadan practitioners worldwide support this belief [11]–[14].

However, Ramadan fasting in the Kingdom of Saudi Arabia, unlike other Muslim and Arab countries, is associated with profound changes in sleeping and feeding patterns, with an almost complete reversal of the rest/sleep vs. wake cycle and restriction of food intake to night-time only. We already reported the effects of these life-style changes on glucose homeostasis, as well as insulin and cortisol secretion patterns, the latter characterized by a complete loss of circadian rhythmicity [15]. However, other circadian hormonal and metabolic effects of Ramadan on insulin tissue sensitivity, in particular the adipokines leptin and adiponectin, have been studied little and questions remain. Here we studied Ramadan-related changes of leptin and adiponectin, both of which regulate appetite and affect metabolic pathways and insulin sensitivity, growth hormone (GH), which influences insulin sensitivity, and hsCRP, a measure of systemic smoldering inflammation, as well as associated changes in acute reactants and liver function.

## Subjects and Methods

### Study Design

We employed samples of the population studied in our earlier report described in brief below [15]. The same subjects were studied at different times to avoid variability between groups at baseline and to decrease sample size. Based on laboratory quality control data, and reference ranges for intended measurements, sample size was calculated to avoid type II statistical error [16], and was found to be 18.7. This was increased further to account for drop-outs, and the total number of recruited subjects was 25. The protocol was approved by the Committee on the Ethics of Human Research at the “Faculty of Medicine- King Abdulaziz University”. Twenty-three of the recruited volunteer healthy subjects (18 males, 5 females), aged 18–42 years; completed the study. Written informed consent was obtained in all cases. Volunteers were studied twice, during their regular life (Shaaban) before, and again 10–15 days into fasting period (Ramadan). They were instructed to have meals as usual on the day of testing, and to record their usual sleeping and waking times for the previous three days. This was because changes in quantity, quality or timing of meals, as well as in sleeping pattern, are expected to affect measured parameters, such as circulating concentrations of glucose, insulin, cortisol, leptin and adiponectin. More details about study protocol, demographic, physiological and anthropometric measurements were described in our previous study [15].

Blood samples were drawn twice daily at 9 am (±1 hour) and again twelve hours later. Thus, sample one and three were obtained while fasting (at least 10 hours for sample one and 6–7 hours for sample 3), and samples two and four were obtained 2–5 hours after meals. Separated serum samples were stored at –80°C until measurements were performed.

### Biochemical and Endocrine Assays

The concentrations of the adipokines, adiponectin and leptin in serum samples were assayed at the “Nutrition Research Unit” Laboratory at King Fahd Medical Research Center. Serum adiponectin was measured using “Biovendor human adiponectin ELISA” high sensitivity kit, while serum leptin was assayed using DRG®Leptin ELISA kit. Both assays were solid phase enzyme-linked immunosorbent assay, and were carried out according to the manufacturers’ procedure. Absorbance was measured at 450±10 nm using a microplate reader (Biokit, ELX800- USA).

Serum biochemical and remaining endocrine parameters were assayed in accredited Clinical Chemistry laboratory at the National Guard Hospital, King Abdulaziz Medical City-Jeddah. For serum glucose, liver enzymes, albumin and total protein tests Abbott architect c16000 auto-analyzer (spectrophotometric method) was used. On the same auto-analyzer, the method of immunoturbidimetric determination technique was utilized to measure hsCRP. Insulin measurement was done on Abbott Architect i2000 auto-analyser by using the method of a chemiluminescent microparticle immunoassay (CMIA). Growth hormone was measured by using chemiluminescent immunometric method on immulite 1000 analyzer.

### Statistical Analyses

Analyses were performed using SPSS statistical package version 16. Descriptive statistics, such as mean ± SEM, were calculated for all estimated parameters. Absolute change in the levels of hsCRP (absolute Δ hsCRP) was calculated by subtracting evening from morning levels.

Paired Student t-test, two related sample (Wilcoxon) test were employed for comparison of normally distributed and non-normally distributed parameters, respectively. Skewed parameters were Ln transformed before analysis (i.e. total hsCRP, total GH). Significance was assigned at p<0.05.

## Results

**Figure 1:**
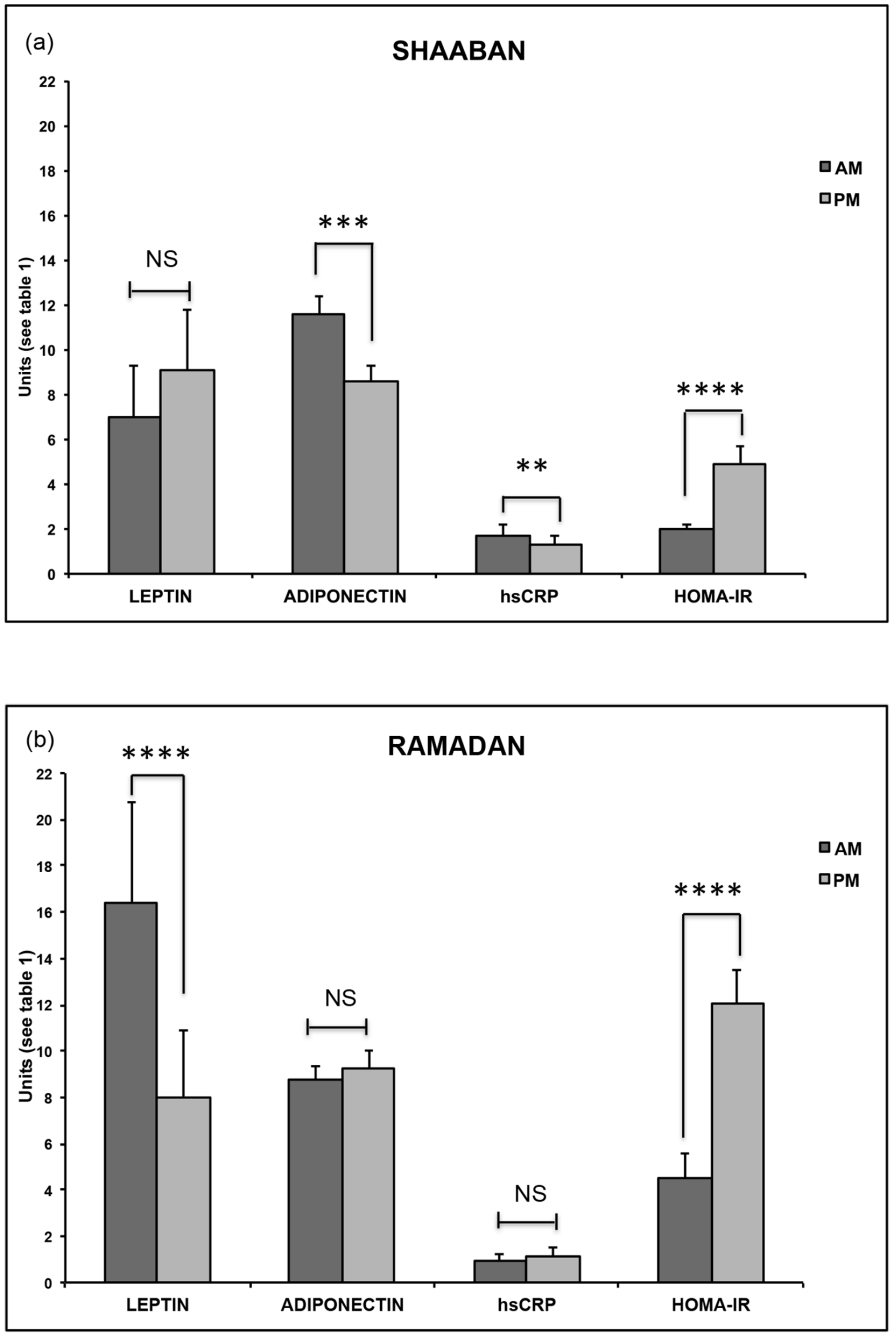
Circulating day and night levels of leptin, adiponectin, hsCRP and HOMA-IR during the Shaaban (a) and 2 weeks into the Ramadan (b) in 23 healthy young Saudi Arabians. **p<0.01, ***p<0.005, ****p<0.0001.

Sleeping and feeding patterns, as well as physiologic, demographic and anthropometric characteristics of studied subjects, were reported earlier [15]. Even though a small change in weight (up to ±0.7 Kg) was noted for most participants, there was no statistically significant change in weight, body mass index (BMI) or percentage body fat during the study period. Results of estimated biochemical and endocrine parameters are presented in Table 1 and figure 1.

**Table 1:** HOMA-IR, and concentrations of adipokines, GH and other metabolic/inflammatory parameters during Shaaban and Ramadan.

		Shaaban		Ramadan		Shaaban vs. Ramadan
		Mean ± SEM	P value (AM-PM)	Mean ± SEM	P value (AM-PM)	P value
**HOMA- IR**	AM	1.98±0.24*	**<0.01**	4.51±1.04*	**<0.01**	0.010*
	PM	4.94±0.80		12.01±1.53		**0.001**
**Leptin (ng/ml)**	AM	7.01±2.27	0.066	16.36±4.35	**0.001**	**0.001**
	PM	9.11±2.66		8.03±2.85		0.451
**Adiponectin (µmol/ml)**	AM	11.62±0.80	**0.001**	8.80±0.57	0.375	**0.001**
	PM	8.61±0.67		9.28±0.78		0.405
**hsCRP (mg/L)**	AM	1.68±0.47	**0.008**	0.97±0.22	0.492	0.089
	PM	1.33±0.32		1.13±0.35		0.476
	Absolute Δ	0.35±0.17	–	0.42±0.17	–	**0.044**
**GH (ng/ml)**	AM	1.72±0.68	0.469	0.85±0.52	**0.028**	0.084
	PM	1.53±0.70		0.18±.06		**0.023**
**TBil (µmol/L)**	AM	6.5±0.7		5.1±0.6		0.153
**GGT (U/L)**	AM	27.6±4.3		22.9±3.9		**0.018**
**AST (U/L)**	AM	20.9±3.1		17.2±1.4		0.199
**ALT (U/L)**	AM	20.7±5.1		17.5±3.5		0.601
**ALKP (U/L)**	AM	79.8±7.0		75.5±6.9		0.199
**Alb (g/L)**	AM	42.0±0.5		40.1±0.4		**0.001**
**TProt (g/L)**	AM	74.7±0.9		71.5±0.7		**0.002**

GH, Growth Hormone; TBil, total Bilirubin; ALKP, alkaline phosphatase; HOMA-IR, homeostasis model assessment of insulin resistance; GGT, gamma glutamyl transferase; AST, aspartate aminotransferase; ALT, alanine aminotransferase; TProt, total protein; hsCRP, high sensitivity C-reactive protein; Alb, albumin; (*), Reported earlier in ref 15, [15].

The mean morning leptin concentration was significantly increased during Ramadan (p = 0.001), in contrast to adiponectin, whose mean concentration was decreased compared to pre- Ramadan (Shaaban) values (p<0.001). These changes in hormonal secretion patterns were associated with increased insulin resistance reflected on a higher HOMA-IR in Ramadan compared to Shaaban both in the morning and evening (p = 0.010 and 0.001, respectively) (Table 1 and figure 1).

The means of growth hormone (GH) concentrations in Ramadan were generally decreased compared to Shaaban, however, only the evening mean GH was significantly lower than the Shaaban mean (p = 0.023) (Table 1). Moreover, the pattern of its secretion at the time of measurements was changed, so that evening mean concentration was significantly lower than the morning mean only during Ramadan (p = 0.028).

Even though the concentrations of the acute phase reactant hsCRP were generally lower during Ramadan than Shaaban, there was a significant circadian change in the mean concentrations only in Shaaban, with hsCRP levels falling in the evening (p = 0.008). No circadian rhythmicity of hsCRP was noted during Ramadan (p = 0.492) (Table 1).

Means of liver enzymes, total bilirubin, and albumin were all lower during Ramadan than Shaaban. However, statistically lower means were only noted for GGT (p = 0.018), total protein (p = 0.002) and albumin (p = 0.001) (Table 1).

## Discussion

Under regular living conditions with normal eating and sleeping patterns, glucose tolerance varies with the time of day, with plasma glucose concentrations being higher in the evening than in the morning [17]. However, as we previously reported, morning glucose concentration was slightly, but not significantly higher than the evening concentration during Ramadan, in spite of a higher insulin concentration, resulting in a higher HOMA-IR [15]. In a series of studies by Spiegel et al. [18]–[20], sleep was reported to have important modulatory effects on glucose regulation, and recurrent sleep loss was associated with marked negative alterations of parameters of glucose tolerance. These findings could explain our reported increased HOMA-IR during Ramadan (Table 1 and figure 1).

The variation in glucose tolerance during the day is partly driven by the diurnal rhythm of plasma cortisol, an important insulin counter-regulatory hormone [21]–[23]. Indeed, the diurnal variation in insulin secretion is inversely related to that of cortisol. Moreover, rises in plasma concentrations of glucose and insulin following short-term elevations of plasma cortisol, are more pronounced in the evening than in the morning, possibly because glucocorticoid sensitivity is increased in the evening hours [3], [23], [24]. We previously reported changes in cortisol secretory patterns during the fasting month of Ramadan, with lack of a decrease in the evening, and suggested that this could be the cause of increased insulin resistance during the fasting month [15]. However, other influences on insulin sensitivity were suspected, in particular those of leptin, adiponectin and GH. The two adipokines increase insulin tissue sensitivity [25]–[28], while GH is an insulin counter-regulatory hormone.

Decreased glucose tolerance during the first part of the night is believed to be due to decreased glucose utilization both by the brain and peripheral tissues - resulting from muscle relaxation and rapid insulin-like effects of sleep-onset GH secretion [29]–[31]. During the second part of the night, these effects subside as sleep becomes shallow and more fragmented and GH is no longer secreted. Thus, complex interactions of circadian and sleep effects, possibly partly mediated by cortisol and GH, result in a consistent pattern of changes of set-point of glucose regulation over the 24-hour period. Fasting was reported to enhance GH release through combined frequency (discrete pulses) and amplitude (sinusoidal periodicities) modulation [32]. However, we found decreased baseline concentrations during the month of Ramadan, as well as a changed secretion pattern (Table 1). This is most likely due to differences in sleeping and meal patterns, and stimulation by food, and is not likely to contribute to the noted insulin resistance. Of course we cannot rule out changes in GH secretory pulses that occur during the first part of sleep, which we would have missed. It is well known that GH increased with exercise [33]. Hence, the less exercise exerted by participants during the fasting month of Ramadan compared to Shaaban, could also contribute to noted changes.

Normally, serum leptin concentrations in healthy individuals show a diurnal rhythm, with nadir values during the daytime, and a nocturnal rise with zenith values during early to mid-sleep [34]. Thus, the diurnal variation of leptin is an approximate mirror image to that of cortisol [35]–[38]. Hence, in fully rested subjects, minimum cortisol concentrations coincide with maximum leptin concentrations [19]. However, the timing of the daily maximum of plasma leptin concentrations is mainly dependent on the timing of meals, with shifts in meal timings inducing immediate shifts in leptin secretory profiles, so that fasting and eating are associated with a decrease and an increase in leptin concentrations, respectively [39]. On the other hand, adiponectin concentrations exhibit ultradian pulsatility, as well as diurnal variation, in healthy subjects, with a significant nocturnal decline, reaching minimum values in the early morning [40]. Thus, the changes in leptin and adiponectin concentrations observed in Ramadan (Table 1 and figure 1) can be explained by the altered sleeping/feeding pattern during this time, as well as by the previously reported changes in cortisol secretion [15]. Furthermore, the significant changes in the secretion of the two adipokines to a pattern favoring insulin resistance, could have augmented the effects of the altered cortisol pattern, leading to a significantly increased of insulin resistance for AM and PM (Table 1).

Another hormone, which plays a major role in glucose homeostasis, is glucagon. Feeding and the biological clock control 24-h plasma glucagon concentrations, and in fed rats, glucagon is not responsible for the daily glucose rhythm, however, during fasting, it may contribute to energy mobilization when the activity period starts [41]. A study demonstrated that during nighttime sleep in healthy non-diabetic subjects, there is a continuous decline of plasma glucagon levels independently of circulating glucose and insulin concentrations [42]. Thus, altered sleep and feeding pattern during the fasting month of Ramadan might have caused a change in glucagon pattern of secretion to one favoring insulin resistance. Unfortunately glucagon was not estimated in our study; therefore no conclusion can be reached.

Intermittent fasting (IF) with adequate sleep has been reported to increase lifespan, even when there is little or no overall decrease in calorie intake [43], [44]. This was attributed to 1. increased insulin sensitivity that results in reduced plasma glucose and insulin concentrations and improved glucose tolerance [44], [45]; 2. reduced levels of oxidative stress, as indicated by decreased oxidative damage to proteins, lipids and DNA [46]; 3. increased resistance to various types of stress, including heat, oxidative and metabolic stresses [47]; and 4. enhanced immune function.

Studies on Ramadan-type fasting in practicing Muslims have given conflicting data, with some reporting beneficial effects [11], [12], [48] while others reporting deleterious effects [49]–[51] on metabolic and hormonal variables. This could be due to differences in dietary and sleeping patterns and habits in different countries [52]–[54].

Sleep disturbance is associated with increased hsCRP [55]. However, in our study hsCRP values were generally lower in Ramadan than Shaaban. Moreover, the significant decrease in the evening hsCRP during Shaaban reported to increase risk of cardiovascular events [56] was not found in Ramadan, probably reflecting the loss of the cortisol circadian rhythm reported earlier [15], since this inflammatory index is normally stimulated by cortisol [9].

Another protein produced by the liver is albumin, the universal carrier. Even though all estimated concentrations remained within normal range, we noted a small, but statistically significant decrease in its mean concentration during Ramadan (p = 0.001) (Table 1). If change in hydration state is the explanation, an increase rather than a decrease would have been expected in Ramadan, in view of abstinence from drink and food during day time. Furthermore, a change in albumin concentration due to hydration status was ruled out based on our earlier reported electrolyte results [15]. It has been long known that protein synthesis in the body as a whole is sensitive to nutritional status [57]–[59], with greater rates observed in the fed compared with the fasted state. More recently, it was reported that liver protein synthesis was more significantly reduced by fasting than other organs [60]. Furthermore, consumption of the daily food all in one meal distorted the circadian rhythm, particularly when it was taken in the morning, and a morning meal increased the total 24 hour synthesis of protein in liver, whereas an evening meal did not [61]. This would explain our findings of reduced hsCRP, albumin and liver enzymes observed in the Ramadan.

Circadian control of sugar- utilizing components (*Glut2*, glucose-6-phosphatase transport protein 1, pyruvate kinase, glucagon receptor) may constitute the underlying molecular bases of circadian control in glucose uptake and insulin response in rodents [62]. Recently, researchers discovered that circadian rhythms control the activity of many genes implicated in all functions of the liver [63]. Thus, the disturbed circadian rhythm experienced in Ramadan might explain our findings further.

In conclusion, it appears that the intended benefits of fasting during the Ramadan might be offset by the deleterious effects of altered circadian rhythms leading to insulin resistance and, hence, increased cardiometabolic risk. Furthermore, since the measurements were carried out approximately in the middle of the fasting month, and the disturbance in sleep/wake cycle, and feeding patterns carries on into the rest of the month during the holiday period, a more pronounced effect might be expected by the time regular work/school schedule is resumed. This is of special importance in our region, which is associated with high prevalence of diabetes mellitus type 2. The noted elevated insulin resistance might increase the possibility of diabetic ketoacidosis in fasting individuals with type 2 diabetes. Indeed, in a study conducted in 13 Islamic countries (including Saudi Arabia) on 12,243 fasting diabetic individuals a significant increase in the number of severe hyperglycemia episodes with/without ketoacidosis per month was found during Ramadan; interestingly, no increase in such episodes was observed in patients with diabetes mellitus type 1 [64]. Furthermore, since prediabetic individuals usually exhibit increased insulin resistance, this type of Ramadan fasting could deteriorate their insulin sensitivity further, hence accelerating their progression to type 2 diabetes. Finally, since we did not study the additional effects of changing composition of macronutrients in the diet on endocrine and biochemical parameters during Ramadan, any future work should include this.
